# Growth Benefits of Own Mother’s Milk in Preterm Infants Fed Daily Individualized Fortified Human Milk

**DOI:** 10.3390/nu11040772

**Published:** 2019-04-03

**Authors:** Virginie de Halleux, Catherine Pieltain, Thibault Senterre, Frédéric Studzinski, Catheline Kessen, Vincent Rigo, Jacques Rigo

**Affiliations:** 1 Department of Neonatology, University of Liège, CHU and CHR Liège, 4000 Liège, Belgium; catherine.pieltain@chuliege.be (C.P.); thibault.senterre@chuliege.be (T.S.); fstudzinski@chuliege.be (F.S.); vincent.rigo@chuliege.be (V.R.); j.rigo@ulg.ac.be (J.R.); 2 Human Milk Bank of CHR Liège, 4000 Liège, Belgium; biberonnerie@chrcitadelle.be; 3 Baxter Healthcare Corporation, Global Medical Affairs, 1420 Braine-l’Alleud, Belgium

**Keywords:** preterm, growth, human milk, donor milk, own mother’s milk, fortification

## Abstract

The influence of types of human milk (HM)—raw own mother’s milk (OMM), pasteurized OMM, and donor milk (DM)—was evaluated for growth in premature infants fed exclusively HM with controlled nutritional intakes using daily individualized HM fortification (IHMF). Growth and nutritional intakes were prospectively collected in preterm infants (<32 weeks) fed IHMF and compared in infants fed predominantly (≥75%) OMM and DM. The influence of HM types (raw OMM, pasteurized OMM, and DM) on growth were also evaluated in the whole population. One-hundred and one preterm infants (birth weight 970 ± 255 g, gestational age 27.8 ± 1.9 weeks) were included. Energy (143 ± 8 vs. 141 ± 6 kcal/kg/day; *p* = 0.15) and protein intakes (4.17 ± 0.15 vs. 4.15 ± 0.14 g/kg/day; *p* = 0.51) were similar in both groups. Infants receiving predominantly OMM (*n* = 37), gained significantly more weight (19.8 ± 2.0 vs. 18.2 ± 2.2 g/kg/day; *p* = 0.002) and length (1.17 ± 0.26 vs. 0.99 ± 0.36 cm/week; *p* = 0.020) than those fed predominantly DM (*n* = 33). Stepwise multivariate analysis (*n* = 101) suggests that raw OMM was the major determinant of growth, contributing 22.7% of weight gain. Length gain was also related to OMM (raw + pasteurized) intakes, explaining 4.0% of length gain. In conclusion, at daily controlled similar protein and energy intakes, OMM had significant beneficial effects on weight and length versus DM in VLBW infants. This difference could be partially explained by the use of raw OMM.

## 1. Introduction

In premature infants, human milk (HM) is associated with significant benefits on health and development. The mother’s own milk (OMM) is therefore always recommended as the first nutritional choice. When OMM is unavailable, the use of donor milk (DM) rather than formula could be the first alternative for very low birth weight (VLBW) infants of less than 32 weeks [1,2,3].

Preterm infants have high nutritional requirements, [4,5,6] and exclusive HM, even from infant’s OMM or banked DM, will not provide intakes that reach current nutritional recommendations. Fortification is therefore recommended to improve post-natal growth [7,8]. Nevertheless, the use of fortified HM could still fail to obtain qualitative and quantitative postnatal growth in the range of fetal growth [9,10,11]. That remains a concern as postnatal nutritional deficit and growth restriction during the neonatal period could be linked to altered long term health and neurodevelopment outcomes [12,13,14] in spite of the beneficial advantages associated with the early HM use [15,16,17].

Worldwide, OMM and DM use in neonatal intensive care units (NICUs) increased over the last decade, but without practical and clear nutritional recommendations [18]. Recent studies, using infrared method, demonstrated the wide variability of protein and energy contents of either DM or OMM, suggesting that the use of theoretical composition values could induce nutritional deficiency or overload [19]. Studies of the impact of individualized HM fortification versus targeted or standard fortification on growth of VLBW infants are scarce [20,21,22,23]. In addition, nutritional interests of fortified raw OMM versus pasteurized OMM or DM are still controversial [10,24,25,26,27]. A few studies have showed lower growth rates in infants receiving fortified DM compared to fortified OMM [25,26,28]. One frequently suggested explanation was the lower protein and fat content of DM frequently provided by mothers who delivered term infants or were in later stages of lactation [29]. Another explanation could be a reduction of the nutrient contents or bioavailability with the processing of DM [27,30].

The primary objective of the present study is to evaluate growth in VLBW infants fed individualized fortified HM with predominant OMM (≥75%) or predominant DM (≥75%). We hypothesized that, using individualized fortification providing controlled similar protein and energy intakes, the use of OMM could improve growth during the early weeks of life. The secondary objective is to determine the influence of raw versus pasteurized OMM, hypothesizing that pasteurization could impair nutrients’ bioavailability and therefore reduce the neonatal growth rate during the study period.

## 2. Materials and Methods

### 2.1. Study Population and Study Design

This is a single center prospective and non-interventional study conducted in the NICU of the University of Liège, Belgium evaluating growth in preterm infants fed HM with individualized fortification (IHMF). From January 1, 2007 to December 31, 2014, data on HM use, HM composition, and fortification in preterm infants born <32 weeks gestation (GA) were collected daily in our NICU human milk bank. From those datasets, preterm infants receiving IHMF as previously reported [19,20] were included in the present study. Infants with chromosomal or congenital anomalies impacting growth and those receiving IHMF for less than 14 days where excluded. 

To evaluate the respective influences of OMM and DM, growth and nutritional intakes (mean ± standard deviation (SD), during the study period, were compared in preterm infants fed predominantly OMM (≥75%) or predominantly DM (≥75%). In addition, the effects of HM types on growth during the study period were evaluated on the whole population, including a third group receiving a mixed HM diet ranging from 26% to 74% of OMM. Under existing Belgian law at the time of the study, the collection of anonymized data concerning clinical routine practices does not require approved of the Ethical Committee. However, the parents were informed and provided consent for donor milk use as necessary, as well as HM analysis and individualized fortification.

### 2.2. Nutritional Practices

Global nutritional management was previously reported [31]. According to our protocol, all VLBW infants received parenteral nutrition on the first day of life with a balanced standardized parenteral solution, designed to provide preterm infants a mean intake of 37–38 kcal/kg/day and 2.4–2.5 g/kg/day of protein on the first day of life followed by a rapid increase to a target intake of 3.8 kcal/kg/day of protein and 120 kcal/kg/day by 5 to 8 days of life [31]. Insulin therapy was only used in case of hyperglycemia (>10 mmol/L) during parenteral nutrition. Enteral nutrition (10–20 mL/kg/day) was initiated within the first hours of life with maternal colostrum or unfortified DM and progressively increased by 10 to 20 mL/kg/day until 160 to 180 mL/kg/day according to tolerance. Mothers were encouraged to breastfeed and received support from dedicated nurses in the unit. HM was expressed at the hospital or at home, by manual expression or by using an electric pump, and transported under aseptic HACCP (Hazard Analysis Critical Control Point) conditions, and mothers were provided with written instructions regarding mechanical expression, milk collection, storage, and transport. OMM provided by the mother was kept at 4 °C and used within 72 h. DM was obtained from our own NICU HM Bank. Milk donors were unpaid volunteers. Informed consent for the use of their milk for feeding preterm infants or for research purposes was obtained Most of these donors had delivered preterm. DM from the early stage of lactation (first week) was separately pooled, processed, labeled, and used during the first days of life in extremely preterm infants in the absence or as a supplement of OMM. DM was always Holder pasteurized (62.5 °C for 30 min) in batches of 5 L. OMM was used as previously described. OMM of cytomegalovirus positive mothers of infants of less than 32 weeks GA at birth was pasteurized until postconceptional age of 34 weeks. A bacteriologic count of OMM was performed after 24 h of incubation, allowing heavy contaminated OMM to be discarded or to use it directly as raw milk or pasteurized milk in case of light contamination [32,33]. Supplemental parenteral nutrition was withdrawn when enteral intakes reached 100 to 120 mL/kg/day. Standard HM fortification was introduced at 25% (addition of 0.275 g of protein and 3.5 kcal in 100 mL of HM) of full fortification once preterm infants tolerated a minimum of 50 mL/kg/d enterally and was gradually increased to full fortification (addition of 1.1 g of protein and 14 kcal in 100 mL of HM). IHMF was considered when a minimum of 140 to 150 mL/kg/day was provided. As IHMF requires extra workload for the HM Bank, its prescription was left to the attending neonatologist. 

### 2.3. Individualized HM Fortification (IHMF)

Fortified HM was prepared daily in the HM Bank. To allow individualized fortification, a sample of 10 mL of HM was taken from the daily pool. Macronutrient HM concentration was determined using a mid-infrared analyzer (Milkoscan minor^®^, Foss, Hillerød, Denmark) previously validated for HM [19]. The Milkoscan analyzer was calibrated to provide the total protein concentration of HM similar to the total nitrogen content, including non-protein nitrogen, measured by a chemical method. HM was warmed to 37 °C and homogenized using an ultrasonic homogenizer (Sonicator^®^, Uppsala, Sweden) before analysis. Data of protein and fat contents were gathered in an excel table to calculate the needs of supplementation according to recommendations [5]. IHMF was performed in two steps: (1) Adjustment of fat content up to 4 g/dL by adding medium-chain triglycerides (MCTs; Liquigen^®^ Danone, The Netherlands), (2) addition of a multicomponent powdered HM fortifier (Enfamil Human Milk Fortifier powder; Mead-Johnson or Nutrilon B.M.F.; Nutricia) to finally provide 4.3 g/kg/day of protein according to the daily volume order. 

### 2.4. Data Collection and Growth Assessment

Day 1 of the study was defined as the first day of IHMF. Weight, HM type (raw OMM, pasteurized OMM, and pasteurized DM), macronutrient composition of HM, MCTs, and fortifier addition and volume intakes were prospectively collected daily during all the IHMF period and used to calculate the nutritional intakes. The energy content was calculated using the Atwater factors: 4 kcal/g for protein and carbohydrate and 9 kcal/g for fat.

Other clinical and demographic data were collected from the medical charts of infants, and this included prenatal complications, delivery information, and neonatal outcomes in the NICU until discharge or transfer to another hospital. 

Infants weight (to the nearest 1 g) was measured daily by nurses using a calibrated electronic scale. Length and head circumference (HC) were assessed weekly (both to the nearest 0.1 cm), length using a length board and HC using a non-stretch measuring tape. Weight gain velocity (grams per kilogram per day) was calculated during the IHMF period using the 2-point average method [34].

$$\text{Weight gain}=\frac{1000*\left(W2-W1\right)}{\frac{W1+W2}{2}*\left(d2-d1\right)}$$

where W = weight in grams; d = day; 1 = beginning of the time interval; and 2 = end of the time interval.

Weight for age, length for age, and head circumference for age Z scores were calculated using Fenton reference growth charts according to corrected GA [35].

### 2.5. Statistical Analysis

Normally distributed data are reported as a mean with standard deviation and groups are compared by using *t*-tests or one-way analysis of variance (ANOVA) with Bonferroni’s correction for post hoc pairwise comparisons. Non-normally distributed data are presented as a median with a range, and groups were compared by Kruskall-Wallis ANOVA tests. Categorical data are presented as numbers and percentages and groups were compared by Chi-squared tests. A *p*-value of <0.05 was considered as significant.

Stepwise multivariate analysis was performed to evaluate the respective influences of significant univariate variables and type of HM (raw OMM, pasteurized OMM, and DM) on growth parameters during the study period. The relation was presented by Pearson correlation coefficient (r or r²). A *p* < 0.05 was considered as significant.

All statistical analyses were performed by using Tibco Statistica software version 13 (TIBCO, Palo Alto, CA, USA). 

## 3. Results

### 3.1. Study Population

Between January 1, 2007 and December 31, 2014, 726 infants with gestational age of less than 32 weeks were admitted to the University of Liège NICU by birth or transfer, of which 665 were discharged alive. The total number of infants that received IHMF during NICU hospitalization was 204. Eighty-two were excluded as they received IHMF of less than 14 days, 12 for chromosomal or congenital anomalies impacting growth, and 9 for incomplete data, leaving 101 subjects included in the study.

### 3.2. Clinical Variables

Out of 101 preterm infants (BW 975 ± 255 g for a GA of 27.8 ± 1.9 weeks), IHMF was initiated at 19 ± 8 days of life during 26 ± 8 days. Thirty-seven infants were fed ≥75% of intake with OMM, 33 infants were fed ≥75% of intake with DM, and 31 with a mixed HM diet with (26%–74% OMM). Demographic and clinical characteristics according to the three HM diets (≥75% OMM versus ≥75% DM versus 26%–74% OMM) are detailed in Table 1. Demographic parameters at birth were similar in the three groups with the exception of HC being significantly lower in the DM group compared to those fed the mixed HM diet.

Neonatal morbidities at study baseline were also similar in the three groups (Table S1) with a trend to a higher incidence of late onset sepsis in the DM group (*p* = 0.062). However, no other significant difference in morbidities that could influence growth was reported between the three groups during and after the study period (Table S2). Necrotizing enterocolitis was observed in three infants, two in the DM group after the study period, (two days after the introduction of preterm formula and the day before suggested discharge in a preterm infant fed formula for several weeks), and the last one in the intermediate group, during the study period, the day after a transfusion. Two infants in the DM category, one in OMM and three in the intermediate group presented clinical infection during or after the study: Five respiratory infections and one urinary tract infection. Insulin treatment rate was similar in all the groups and was only used in case of hyperglycemia during parenteral nutrition. No infants received insulin during the study period.

### 3.3. Influence of OMM Versus DM

According to the primary objective of the study, nutritional intakes and growth during IHMF were compared in VLBW infants fed predominantly OMM and DM.

#### 3.3.1. Human Milk Composition and Nutritional Intakes

The contributions of the HM categories in the two groups are gathered in Table 2. OMM accounted for, respectively, 95.4% and 2.2% of the HM intakes during the IHMF study. Lipid content was significantly higher in the OMM than in the DM group. Nevertheless, in both groups, fortified HM provided similar mean energy and protein intakes with low variability, accounting for, respectively, less than 5.6% and 3.6 % for energy and 3.6% and 3.4% for protein. 

#### 3.3.2. Growth

As shown in Table 3, weight (*p* = 0.002) and length gain (*p* = 0.020), but not HC gain (*p* = 0.120), were significantly higher in infants receiving predominantly OMM compared to those fed predominantly DM during the IHMF period. Similarly, Z-scores gains for weight (*p* < 0.0001), length (*p* = 0.004), and HC (*p* = 0.013) were all significantly higher in infants receiving mostly OMM than in those fed mostly DM during the IHMF period. 

### 3.4. Effects of Type of Human Milk (Raw OMM, Pasteurized OMM, and Pasteurized DM)

#### 3.4.1. Human Milk Composition and Nutritional Intakes

In line with the secondary objective of the study, the whole population was evaluated according to the main HM type received during the study period, DM > 50% (DM), DM ≤ 50%, pasteurized > raw OMM (POMM), and DM ≤ 50% and raw > pasteurized OMM (ROMM) to evaluate the influence of OMM pasteurization on growth velocity during the study period. As shown in Table 4, DM accounted to 88.5% in the DM group (*n* = 45), pasteurized OMM to 70.3% in the POMM group (*n* = 41), and raw OMM to 69.1% in the ROMM group (*n* = 15). Energy and protein intakes during the study period were similar in the three groups.

#### 3.4.2. Growth

Both weight gain and weight Z-score gain in the DM group were significantly lower than in the other two groups. In addition, weight gain, but not weight Z-score gain, was significantly higher in the ROMM versus POMM group. Length and HC gains were similar in the three groups. Nevertheless, the length and HC Z-score gains were significantly improved in the ROMM group compared to the DM group.

### 3.5. Univariate and Multivariate Analysis on the Whole Population

#### 3.5.1. Univariate Analysis

Univariate linear regression analysis on the whole population, showed that birthweight, gestational age, postnatal age at study day 1, as well as protein and energy intakes did not significantly influence weight and length gain during the study period.

Weight gain during the IHMF period was significantly influenced by two univariate factors; study duration (r = 0.31, *p* = 0.0014) and percentage of raw OMM (r = 0.47, *p* < 0.00001). For length, the percentage of total OMM (r = 0.20, *p* = 0.046) was the only factor significantly influencing length gain. 

#### 3.5.2. Multivariate Analysis

##### *Weight Gain and Weight for Age Z-score Difference* 

Stepwise multivariate analysis demonstrated that weight gain (g/kg/day) was positively related to the proportion of raw OMM, proportion of pasteurized OMM, and postnatal age at the first day of study, but negatively related to study duration and birthweight. Those factors explain 22.7%, 3.7%, 3.1%, 9.8%, and 3.0% of the weight gain, respectively. It was also estimated that the weight for age Z-score difference during IHMF was related to the raw OMM proportion, gestational age, and birth weight, contributing, respectively, to 18.0%, 12.1%, and 10.7% of the Z-score difference.

##### *Length Gain and Length for Age Z-score Difference* 

For length gain, only two parameters were significant; the proportion of total OMM (raw + pasteurized) and postnatal age at baseline, explaining, respectively, 4.0% and 4.4% of the length gain. Similarly, length for age Z-score difference was related to the proportion of total OMM (raw + pasteurized) and study duration, contributing, respectively, to 6.5% and 5.4% of the difference. 

## 4. Discussion

This study is the first providing daily controlled nutritional intakes in preterm infants fed HM with individualized fortification after daily determination of HM composition by a validated infrared method [19,20]. Because of IHMF, protein and energy intakes were similar with very low variability (Table 2) in the two groups, it adequately allows for comparisons of growth and metabolic tolerance in VLBW infants fed exclusively fortified OMM (95.4% ± 7.8%) or DM (97.8% ± 5.4%). This study found that weight gain velocity during IHMF was on average 1.6 g/kg/d higher in infants fed OMM than in those fed DM, with an additional benefit on length gain of around 0.18 cm/week on average, suggesting a growth specific effect of OMM in preterm infants. In addition, the use of predominant OMM (≥75%) instead of predominant DM (≥75%) significantly improved weight, length, and HC Z-score changes during the study period (Table 3).

As shown in Table 3, around two thirds of OMM was provided after Holder pasteurization and not as raw OMM. This is mostly explained by the strategy applied to reduce the risk of infectious transmission with raw milk. According to our previous study [32,33], up to 20%–50% of the OMM samples were contaminated and were either pasteurized or discarded. In addition, to avoid CMV contamination or infection [30], OMM of CMV seropositive mothers of VLBW infants was also systematically pasteurized. The variability of the raw OMM intakes in our whole population allowed us to evaluate the respective role of raw OMM versus pasteurized OMM or DM on growth velocity in the preterm infants. We found that ROMM and POMM both have a positive effect on weight gain, contributing to an increase of +2.8 g/kg/day and +0.9 g/kg/day, respectively, compared to DM. It suggests that the major positive effect of OMM could be the result of its use as a raw product, with a mean weight gain difference of 2.0 g/kg/day compared to pasteurized OMM (Table 4). Our study also suggests that the use of raw OMM also induces a significant positive effect on weight (*p* = 0.003), length (*p* = 0.013), and HC (*p* = 0.016) Z-score gains during the study period compared to DM. The benefits of POMM on DM was limited on weight gain and weight Z-score gain whereas benefits on length and HC Z-scores were not significant with *p* values of 0.2 and 0.07, respectively, contrasting with the benefits observed with ROMM. Therefore, our study suggests that the limited beneficial effect of POMM versus DM remains to be confirmed in additional studies. 

**Figure 1:**
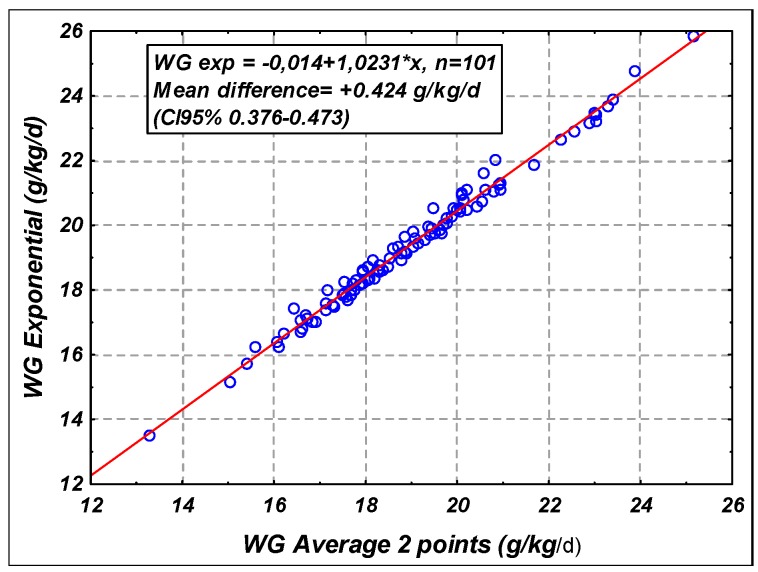
Evaluation of growth velocity by the exponential and the average 2 points methods.

The optimal reference growth chart to evaluate postnatal growth velocity in preterm infants is still debated as discussed recently by an international expert group [34]. From this review, it was recommended to use the average 2 points or the exponential 2 points methods to evaluate the growth velocity. Both formulas provide similar results that are highly correlated with a slightly higher value for the exponential method as shown in the Figure 1. In our study, we chose to use the average 2 points method for comparison to our previous studies [9,20,31,36].

In addition, this review and others [34,37] suggest that it is time to report growth studies in a standardized fashion. The standardized growth report is also debated and several growth charts have been proposed to evaluate postnatal Z-scores in VLBW infants. Recently, it was suggested that the Fenton revised growth charts of 2013 could be outdated by the recent INTERGROWTH-21st Postnatal Follow-up Study of preterm infants [38]

We agree that both the Fenton growth chart and the INTERGROWTH-21st have some limitations. The Fenton growth charts are built with a meta-analysis of cross-sectional fetal charts without take into account that postnatal growth composition differs to that of fetal growth composition. By contrast, the INTERGROWTH-21st chart is longitudinal, not cross-sectional and non-fetal. However, this contains some limitations, including the small number of very preterm infants included in the database, as well as the lack of “gold standard nutrition” [39,40]. Indeed, the description of the feeding regimen in the INTERGROWTH study is limited. It is specified that the main feeding regimen of the included preterm infants was human milk and that the use of HMF was only added to expressed HM until a baby’s weight reached 1800–2000 g and not up to discharge. The daily protein and energy intakes were not adequately controlled during the postnatal period, suggesting that some cumulative protein and energy deficits could induce relative postnatal growth restriction in the evaluated population of preterm infants. The authors of the INTERGROWTH-21 group [39] recommend the INTERGROWTH-21st Preterm Postnatal Growth Standards for monitoring the growth of more than 90% of preterm infants who are born at ≥32 weeks and recognize that the construction of charts for very preterm infants (<32 weeks’ gestation) is problematic. We consider that our population, including 30% of preterm infants with a GA < 27 weeks and 100% at <32 weeks at birth, but also 80% still <32 weeks at baseline, is not in the optimal range of the INTERGROWTH reference. Therefore, our results were compared to the combined references growth chart of the fetus and the term infants as proposed by Fenton et al. in 2013 [35]. However, data of preterm infants >27 weeks GA were also compared to the INTERGROWTH-21st reference in Figures S1 and S2.

This study demonstrates a significant positive impact of both OMM and raw OMM on growth in preterm infants fed HM. This effect seems independent of nitrogen, lipid, and carbohydrate content as this was controlled by the IHMF in this study. Nutritional and growth benefits of fortified OMM versus fortified DM is still debated and studies report controversial results regarding growth and Z-score changes in preterm infants. Thus, in two observational and one retrospective study, a weight gain benefit was reported in preterm infants fed fortified OMM. In 2011, Montjaux et al. [25] suggested that weight gain was directly proportional to the amount of fresh raw OMM compared to pasteurized fortified DM (*n* = 48). More recently, Madore et al. [26] showed a significantly higher weight gain in preterm infants fed predominantly fortified OMM compared to those fed predominantly fortified DM during the first month of life (*n* = 56). Brownell et al. [28], using OMM as a reference, also reported a significant decrease in mean weight and head velocity during a hospital stay for every 10% increase of the total feeding volume provided as DM (*n* = 314). By contrast, two retrospective studies did not observe any significant difference in weight gain between premature infants receiving either exclusively OMM or DM as a sole diet (*n* = 92) [41] or in those fed predominantly (>50%) fortified OMM or fortified DM (*n* = 299) [42]. In addition, a third retrospective study found no significant difference in weight Z-score change by HM diet (*n* = 88) (>75% donor vs. >75% OMM; *p* = 0.28) [10]. In contrast to our study, none of those studies precisely determined and controlled the protein and energy intakes, and the rate of pasteurization, if any, in the OMM groups was not specified. Still, the effect of pasteurization on growth velocity was recently evaluated as a secondary outcome in a randomized study of more than 300 premature infants receiving fortified OMM either raw or pasteurized. In that study, a similar growth was observed between the two groups [43]. 

In preterm infants fed fortified HM, postnatal growth restriction was frequently reported as well as loss of Z-score during the full HM fortification period [10,44]. Repetitively, recommendations from various expert committees suggest that nutritional requirements are similar in VLBW infants fed fortified HM or preterm formula (PTF) [4,6]. Until now, no specific guidelines have been proposed for fortified HM fed preterm infants. However, it is recognized that at similar controlled protein and energy intakes, growth velocity is significantly lower in preterm infants fed fortified HM than in those fed PTF [9]. Metabolic and energy balance studies show that such a difference could be the result of lower metabolized protein and energy contents of fortified HM compared to PTF [36]. The mean difference in nitrogen utilization (retention/intake) as well as the mean difference in energy absorption rates measured by bomb calorimetry were both about 10% less with fortified HM [45]. This difference could be partially due to the use of pasteurization. In addition, as shown more recently, the use of standard reference values for OMM and DM may induce an overestimation of the protein and energy content of fortified HM [19,46]. While preterm OMM with its higher protein content could improve growth compared to DM, it remains insufficient to support adequate growth, especially after the first month of lactation when the OMM protein concentration decreases [29]. A previous study performed in our NICU found that the macronutrient and energy content of OMM was highly variable and unpredictable. Protein and energy content of DM was also significantly lower than that of OMM [19]. Of all the daily OMM and DM samples (*n* = 2630) measured in the present study, 67% were <1.5 g protein/dL and 62% were <67 kcal energy/dL, values commonly considered as reference values for HM composition to estimate nutrient intakes in clinical practice.

By using metabolic balance studies and indirect calorimetry, we previously showed that protein intake and the protein energy ratio were major determinants of weight gain in VLBW infants [36]. In a recent multicentric study [46], we showed that theoretical intakes of 4.46 g/kg/day of protein and 125 kcal/kg/day (not confirmed by HM content analysis) led to a stable weight Z-score during the study period in VLBW infants receiving new HMF while the weight Z-score decreased significantly in the control HMF group theoretically receiving 3.81 g/kg/day of protein and 125 kcal/kg/day. Trends in the same direction were observed for length Z-score changes. In that study, the protein intakes were not measured, but estimated according to a preterm HM reference [47] and were therefore probably overestimated in regard to the large use of DM and pasteurized OMM [46]. Based on blood urea nitrogen and urinary urea excretion, we speculated that protein utilization in the new HMF might not have been optimal due to a relative deficiency in metabolized energy intake [46].

Considering the variability of HM macronutrient contents and the lower bioavailability of HM, in the present study, we targeted higher mean protein and energy intakes than those generally recommended [4,6,48]. Thus, during the study period, preterm infants received controlled mean intakes of 143 kcal/kg/day and 4.2 g/kg/day of proteins between 30.5 and 34 weeks’ post-menstrual age, resulting in mean positive weight and HC Z-scores changes of 0.13 and 0.59, respectively, but a limited negative mean length Z-score change of 0.25 in preterm infants fed ≥75% OMM. By contrast, negative Z-score changes for weight (0.26 on average) and length (0.59 on average) were observed in the group receiving ≥75% DM (Table 3). These results suggest that such intakes are close to the minimal requirements necessary for preterm infants fed fortified OMM in such a range of post-menstrual age, but could still be limited in those fed fortified pasteurized DM. In addition, knowing that postnatal growth quality differs to that of fetal growth by an increase in fat deposition, the discrepancies between weight and length Z-scores benefits could be the result of a relative deficit in the lean body mass accretion rate during the study period. Therefore, our study also suggests that protein and energy requirements of preterm infants fed fortified HM are higher than that currently recommended [4,6,48] and that specific nutritional guidelines for HM fed preterm infants need to be designed, promoting the use of OMM, but considering the limitations of its use as raw OMM in VLBW infants.

Improving HM fortification by IHMF through the use of infrared technology and extra protein and energy supplementation may be one of the strategies to optimize the nutritional composition of HM to meet the nutritional needs of preterm infants, especially when DM is used. It was demonstrated that IHMF decreases the variability linked to HM content and safely optimizes protein and energy intake [19,21,49,50]. Premature infants fed with low macronutrient content HM benefit the most from IHMF, with improved growth outcomes. However, infrared devices, originally developed for use in the dairy industry, must be calibrated and validated for HM analysis before clinical use by following good laboratory and clinical practice, and appropriate sample preparation must be done otherwise their use can affect the growth outcomes of preterm infants [19,50,51]. 

## 5. Conclusions

Our study is one of the first studies showing that a daily controlled high protein and energy intakes (4.2 g of protein and 143 Kcal/kg/day) of fortified raw OMM is associated with important growth benefits in preterm infants. It also suggests that pasteurized OMM provides a limited, but significant growth benefit compared to DM, suggesting that pasteurization significantly impaired the bioavailability of protein and energy intake. The increase in protein and/or energy intakes in preterm infants receiving fortified pasteurized HM could be postulated in view of these results, but needs to be demonstrated in further studies. In addition, our study also suggests that specific and different nutritional recommendations need to be designed for preterm infants fed OMM and DM. 

## Figures and Tables

**Table 1:** Infants clinical characteristics according to human milk diet.

m ± SD	≥75% OMM *n* = 37	26%–74% OMM *n* = 31	≥75% DM *n* = 33	All Subjects *n* = 101	*p*
Male sex, n (%)	18 (49)	15 (48)	17 (52)	50 (50)	0.96
Gestational age, weeks,	27.7 ± 2.1	28.2 ± 1.9	27.5 ± 1.8	27.8 ±1.9	0.26
Birth Weight, g,	983 ± 244	1042 ± 312	901 ± 185	975 ± 255	0.08
Birth Weight < 1000 g, n (%)	20 (54)	16 (52)	24 (73)	60 (59)	0.16
Mean Weight z score,	−0.19 ± 0.99	−0.37 ± 0.89	−0.48 ± 0.82	−0.34 ± 0.91	0.47
Birth Length, cm,	35.0 ± 3.3	35.8 ± 3.9	34.6 ± 2.9	35.1 ± 3.4	0.34
Birth HC, cm,	24.9 ± 1.9	25.8 ± 2.3	24.5 ± 1.7	25.0 ± 2.0	0.02
Vaginal Delivery, n (%)	16 (43)	9 (29)	7 (21)	32 (32)	0.13
Twin, n (%)	8 (22)	12 (39)	6 (18)	26 (26)	0.13
Apgar Score 1 min,	6.5 ± 2.2	6.1 ± 2.2	6.1 ± 2.0	6.2 ± 2.1	0.60
Apgar Score 5 min,	7.9 ± 1.5	7.8 ± 1.5	7.9 ± 1.1	7.9 ± 1.4	0.92
Antenatal steroids, n (%)	35 (95)	27 (87)	29 (88)	91 (90)	0.30
Study duration,	27 ± 8	27 ± 8	24 ± 6	26 ± 8	0.14
GA age at study day 1, weeks,	30.5 ± 1,5	30.8 ± 1,6	30.5 ± 1,5	30,6 ± 1,5	0.64
GA age at study end, weeks,	34.2 ± 1.4	34.7 ± 1.8	33.9 ± 1.5	34.3 ± 1.6	0.12

OMM = own mother’s milk; DM = donor milk; GA = gestational age; data are presented as n (%) for categorical variables and mean (m) ± standard deviation (SD) for continuous variables; *p* < 0.05 based on ANOVA for continuous variable and chi square for categorical variables.

**Table 2:** Human milk composition and nutritional intakes during study in the two groups.

	≥75% OMM *n* = 37	≥75% DM *n* = 33	*p*-Value
**Human Milk Category (%)**			
Raw OMM	31.3 ± 33.6	0.5 ± 3.0	<0.001
Pasteurized OMM	64.1 ± 33.1	1.7 ± 4.7	<0.001
Pasteurized DM	4.6 ± 7.8	97.8 ± 5.4	<0.001
**Human Milk Composition (Infrared)**			
Protein, g/dL	1.44 ± 0.22	1.35 ± 0.14	0.056
Lipid, g/dL	3.87 ± 0.59	3.61 ± 0.23	0.022
Carbohydrates, g/dL	6.84 ± 0.22	6.86 ± 0.19	0.695
**Nutritional Intakes (Units/kg/day)**			
Volume, mL	167 ± 10	166 ± 8	0.536
Energy, kcal	143 ± 8	141 ± 6	0.148
Protein, g	4.17 ± 0.15	4.15 ± 0.14	0.512

Data are presented as mean ± SD; *p* < 0.05 based on *t*-test.

**Table 3:** Growth rate and Z-score gain in preterm infants fed individualized fortified with predominantly own mother’s milk (OMM) or donor milk (DM.)

	OMM ≥ 75% *n* = 37	DM ≥ 75% *n* = 33	Delta OMM vs. DM	*p*
Weight gain, g/kg/day	19.8 ± 2.0	18.2 ± 2.2	+1.6	0.002
Length gain, cm/week	1.17 ± 0.26	0.99 ± 0.36	+0.18	0.020
Head circumference, cm/week	1.13 ± 0.22	1.04 ± 0.27	+0.09	0.120
Weight Z-score gain, g/kg/d	0.13 ± 0.35	−0.26 ± 0.41	+0.39	<0.001
Length Z-score gain, cm/week	−0.25 ± 0.41	−0.59 ± 0.52	+0.33	0.004
HC Z-score gain, cm/week	0.59 ± 0.50	−0.24 ± 0.65	+0.35	0.013

Data are presented as mean ± standard deviation; *p* < 0,05 based on *t*-test.

**Table 4:** Growth rate and nutritional intakes according to the main human milk type received during the study period.

Human Milk Type Volume Intake (%)	DM 88.5 ± 16.9	POMM 70.3 ± 22.6	Delta vs. DM	*p* vs. DM	ROMM 69.1 ± 19.9	Delta vs. DM	*p* vs. DM	Delta vs. POMM	*p* vs. POMM
***n***	45	41			15				
Energy, kcal/kg/day	141.3 ± 6.3	142.4 ± 7.3	-	0.432	143.7 ± 6.2		0.210	-	0.552
Protein, g/kg/d	4.15 ± 0.14	4.19 ± 0.13	-	0.211	4.18 ± 0.15		0.494	-	0.855
Weight gain, g/kg/d	18.2 ± 1.9	19.1 ± 1.8	+0.87	0.035	21.1 ± 1.6	+2.83	<0.001	+1.96	<0.001
Length gain, cm/week	1.04 ± 0.36	1.13 ± 0.33	+0.10	0.193	1.17 ± 0.28	+0.14	0.194	+0.04	0.697
HC gain, cm/week	1.04 ± 0.24	1.10 ± 0.20	+0.05	0.258	1.10 ± 0.24	+0.06	0.409	+0.01	0.937
Weight Z-score gain	–0.23 ± 0.39	0.09 ± 0.31	+0.31	<0.001	0.15 ± 0.44	+0.38	0.003	+0.06	0.546
Length Z-score gain	–0.53 ± 0.52	–0.36 ± 0.45	+0.17	0.116	–0.14 ± 0.50	+0.39	0.013	+0.22	0.114
HC Z-score gain	0.28 ± 0.59	0.51 ± 0.56	+0.23	0.068	0.70 ± 0.41	+0.41	0.016	+0.18	0.252

DM = donor milk; POMM = pasteurized own mother’s milk; ROMM = raw own mother’s milk. Data are presented as mean ± standard deviation; *p* < 0.05 based on *t*-test.

## Supplementary Materials

The following are available online at https://www.mdpi.com/2072-6643/11/4/772/s1, Table S1: Initial clinical outcomes before daily individualized HM fortification (IHMF); Table S2: Post baseline clinical outcomes. Figure S1. Weight Z-score and Weight Z-score change according to Fenton and Intergrowth during the study in all preterm infants included in the study with a GA > 27 weeks; Figure S2. Weight for age Z-score at day1 and at the end of the study period, and Z-score gain during the study in infants fed mostly donor (*n* = 45) versus raw OMM (*n* = 15). Comparison of FENTON and INTERGROWTH’s references.
