# Supplementary material for: Growth Benefits of Own Mother’s Milk in Preterm Infants Fed Daily Individualized Fortified Human Milk
Source: Nutrients. 2019 Apr 3;11(4):772. doi: 10.3390/nu11040772 (PMC6521225; doi:10.3390/nu11040772)
Supplement: Supplementary file 1 [file nutrients-11-00772-s001.pdf]

Table S1. Initial clinical outcomes before daily individualized HM fortification (IHMF).

	≥75%OMM<br>*n* = 37	26%–74%OMM<br>*n* = 31	≥75%DM<br>*n* = 33	*p*
Time to full enteral feeding, days, m ± SD	14 ± 7	14 ± 8	15 ± 6	0.89
Mechanical ventilation, n (%)	25 (68)	21 (68)	25 (76)	0.71
Duration of ventilation, days, median (IQR)	0 (0–3)	1(0–6)	1(0–6)	0.57
Inotropes, n (%)	6 (16)	3 (10)	6 (18)	0.61
PDA, n (%)	14 (38)	14 (45)	13 (39)	0.82
PDA surgery, n (%)	5 (14)	1 (3)	4 (12)	0.33
IVH ≥ grade 2, n (%)	2 (1)	5 (12)	3 (9)	0.78
Insulin treatment, n (%)	11 (30)	8 (26)	13 (39)	0.43
Early onset sepsis, n (%)	1 (3)	4 (13)	3 (9)	0.29
Proven late onset sepsis, n (%)	7 (19)	3 (10)	11 (33)	0.062

OMM = own mother's milk; DM = donor milk; Data are presented as n (%) for categorical variables and mean ± standard deviation or median (interquartile range) for continuous variable; $p < 0.05$ based on ANOVA for continuous variables and chi square for categorical variables.

Table S2. Post baseline clinical outcomes.

	≥75%OMM<br>*n* = 37	26%–74%OMM<br>*n* = 31	≥75%DM<br>*n* = 33	*p*
Bronchodysplasia grade 2, n	6 (16)	5 (16)	6 (18)	0.98
Late onset sepsis, n	1 (3)	3 (10)	2 (6)	0.49
PVL, n	0 (0)	1 (3)	1 (3)	0.55
ROP ≥ stage 2, n	1 (3)	2 (7)	2 (6)	0.74
Transfusion, n	18 (49)	17 (55)	22 (67)	0.32
Postnatal steroids, n	4 (11)	4 (13)	2 (6)	0.65
NEC bell ≥ stage 2, n	0 (0)	1 (3)	2 (6)	0.33
NEC, surgery, n	0 (0)	0 (0)	1 (3)	0.35
Death, n	0 (0)	0 (0)	1 (3)	0.35

OMM = own mother's milk; DM = donor milk; BPD grade 2 (moderate) on $FiO_2 > 21\%$ at 28 days, and on FiO_2 22%–30% and having failed oxygen reduction test at 36 weeks; Data are presented as n (%) for categorical variables and * $p < 0.05$ based on chi square.

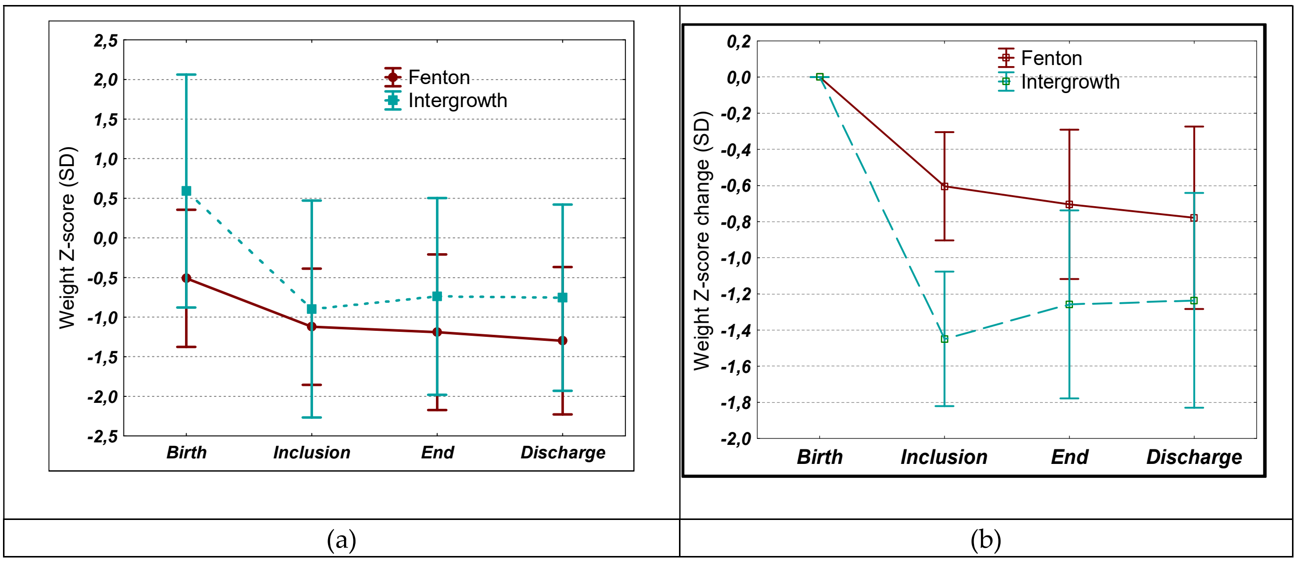

(a)	(b)

Figure S1. Weight Z-score and weight Z-score change according to Fenton and INTERGROWTH during the study in all preterm infants included in the study with a GA > 27 weeks; *n* = 70.

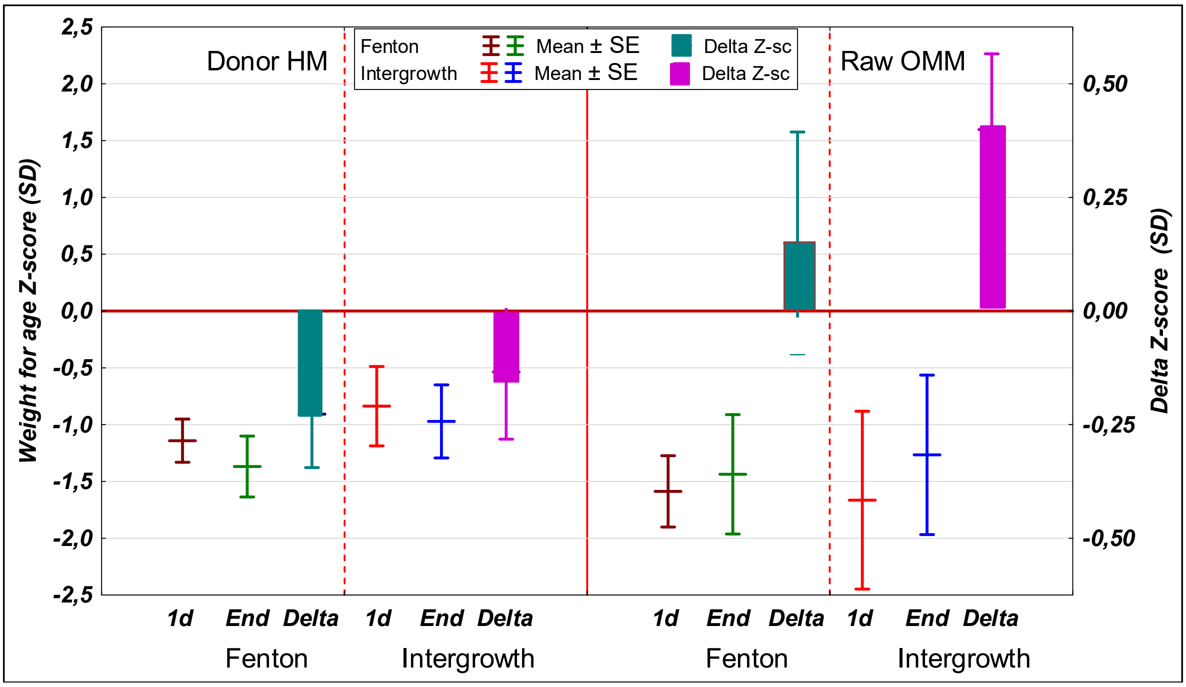

Figure S2. Weight for age Z-score at day1 and at the end of the study period, and Z-score gain during the study in infants fed mostly donor (*n* = 45) versus raw OMM (*n* = 15). Comparison of FENTON and INTERGROWTH references.
